# ﻿Two new species of *Phallus* (Phallaceae) with a white indusium from China

**DOI:** 10.3897/mycokeys.85.75309

**Published:** 2021-12-16

**Authors:** Ting Li, Wang-Qiu Deng, Bin Song, Ming Zhang, Mu Wang, Tai-Hui Li

**Affiliations:** 1 State Key Laboratory of Applied Microbiology Southern China, Guangdong Provincial Key Laboratory of Microbial Culture, Collection and Application, Institute of Microbiology, Guangdong Academy of Sciences, Guangzhou 510070, China Institute of Microbiology, Guangdong Academy of Sciences Guangzhou China; 2 College of Science, Tibet University, Lhasa 850011, China Tibet University Lhasa China; 3 Tibet Agricultural and Animal Husbandry University, Nyingchi 860000, China. Tibet Agricultural and Animal Husbandry University Nyingchi China

**Keywords:** Edible mushrooms, Gasteromycetes, *Phallusindusiatus*, phylogeny, taxonomy

## Abstract

Two new *Phallus* species, *P.cremeo-ochraceus* and *P.rigidiindusiatus* were discovered in southwestern and southern China, respectively. *Phalluscremeo-ochraceus* is morphologically characterized by its cream to ochraceous receptacle, white to very slightly pinkish indusium, white to pinkish pseudostipe and white to slightly purplish pink volva. *Phallusrigidiindusiatus* is characterized by a white to yellowish white receptacle, a strongly rigid indusium usually without serrated margin and smaller basidiospores than those of *P.serratus*. Phylogenetic positions of the two species are located in two independent lineages respectively. Detailed descriptions, color photographs, illustrations and a key to the related species are presented.

## ﻿Introduction

*Phallus* Junius ex L. (1798) is a well-known and widespread gasteroid genus from tropical to temperate zones. Studies based on molecular phylogenetic analyses about a dozen years ago have shown that the existence of an indusium and a perforate pore at top of receptacle has no phylogenetic significance at generic level, and members of *Dictyophora* Desv. (1809), which are mainly characterized by possession of an indusium, should be merged into genus *Phallus* (Cabral et al. 2012; Moreno et al. 2013). In the last decade, quite a lot of species with or without an indusium have been discovered under the genus of *Phallus* (Mohanan 2011; Li et al. 2014; Rebriev et al. 2014; Adamčík et al. 2015; Li et al. 2016; Medeiros et al. 2017; Trierveiler-Pereira et al. 2017; Song et al. 2018; Cabral et al. 2019; Li et al. 2020a).

Thirty-one species, nearly one-third of the world’s total members of known *Phallus* species, have been recorded in China, and sixteen of them were originally reported from there. Many of them are notably edible mushrooms, for instance, *Phallus fragrans* M. Zang, *P.haitangensis* H.L. Li, P.E. Mortimer, J.C. Xu & K.D. Hyde, *P.lutescens* T.H. Li, T. Li & W.Q. Deng and *P.luteus* (Liou & L. Hwang) T. Kasuya; and some have even been produced commercially, e. g. *P.dongsun* T.H. Li, T. Li, Chun Y. Deng, W.Q. Deng & Zhu L. Yang, *P.echinovolvatus* (M. Zang, D.R. Zheng & Z.X. Hu) Kreisel, *P.rubrovolvatus* (M. Zang, D.G. Ji & X.X. Liu) Kreisel and *P.serratus* H. Li Li, L. Ye, P.E. Mortimer, J.C. Xu & K.D. Hyde (Zang end Ji 1985, 1988; Kreisel 1996; Kasuya 2008; Li et al. 2014, 2016, 2020a).

In the past decades, *Phallusindusiatus* Vent. (1798), characterized by a white and touching-ground indusium, had been reported from the tropical and subtropical Africa and Asia, temperate China, Japan, South Pacific islands, Australia and South America (Dring 1964; Kobayasi 1965; Liu et al. 2005; Young 2005; Cabral et al. 2019). However, recent studies revealed that many collections named as “*Phallusindusiatus*” or “*Dictyophoraindusiata* (Vent.) Desv. (1809)” were misidentified, and *P.indusiatus* might be only distributed in Brazil and adjacent countries in South America, rather than widespread from the temperate and subtropical zones (Zang and Ji 1985; Calonge et al. 2005; Song et al. 2018; Cabral et al. 2019). *Phallusindusiatus* s.s. has recently been redescribed with a neotype, which strongly suggested that the *P.indusiatus*-like species from other continents should be considered different taxa from *P.indusiatus* s.s. (Cabral et al. 2019).

During these years, the authors further investigated the diversity of *Phallus* species from China with some new collections. Based on detailed morphological data and DNA-based phylogenetic analyses, two additional new *P.indusiatus*-like species to science were confirmed, and then formally introduced in this study.

## ﻿Materials and methods

### ﻿Morphological studies

Fresh specimens of *Phallus* with white or nearly white indusium were collected from various sites in southern and southwestern China. Photographs of the basidiomata were taken in the field with digital cameras in natural light. Voucher samples were dried with an electronic dryer and deposited in the Fungorum of Guangdong Institute of Microbiology (GDGM), Guangzhou, China. Methods for morphological descriptions followed the previous study by Li et al. (2020a). Color codes mentioned in the description were referenced from Kornerup and Wanscher (1978). Basidiospore dimensions were given as: (a) b–c (d), in which b–c contains 90% of the measured values and a or d represent extreme values. Q denotes to length/width ratio of an individual basidiospore, Q_m_ refers to the average Q value of all basidiospores.

### ﻿Molecular studies

Genomic DNA were extracted from the dried materials using fungi Genomic DNA Purification Kit (Sangon Biotech Co., Ltd.) following the instructions. The nuclear ribosomal large subunit (LSU) and internal transcribed spacer (ITS) regions were amplified using primer pairs LROR/LR5 and ITS1-F/ITS4, respectively (Vilgalys and Hester 1990; White et al. 1990). Newly generated sequences in this study were deposited to GenBank (https://www.ncbi.nlm.nih.gov/genbank). Available sequences of related species of *Phallus* and *Mutinus* were retrieved from the databases of GenBank or Unite Community (https://unite.ut.ee/), whereafter, aligned and edited the matrix of sequences using MAFFT v.7 (Katoh and Standley 2013) and BioEdit v.7.0.9 (Hall 1999).

In order to infer the phylogenetic relationships among new species and other known taxa of *Phallus*, two analyses were run; one for the ITS dataset and the other for ITS and LSU concatenated dataset. Maximum Likelihood (ML) and Bayesian Inference (BI) analyses were performed with MEGA v.7.0 (Hall 2013) and MrBayes v.3.1.2 (Ronquist and Huelsenbeck 2003), respectively. The best substitution model (Tamura 3-parameter+G+I) was chosen for both ITS and concatenated ITS-LSU analyses. Bootstrap (BS) analysis was implemented with 1,000 replicates. BI was calculated with 4 million and 14 million generations for ITS and ITS-LSU datasets, respectively, and stoprule command with the value of stoprule set to 0.01. Trees were sampled every 100 generations and obtained using the sump and sumt commands with the first 25% generations discarded as burn-ins. Branches corresponding to partitions reproduced <50% BS replicates were collapsed; the confidence values of BI were estimated with Posterior probabilities (PP), and discarded the values without reaching 0.95 PP. Trees were edited using FigTree version 1.4.2.

## ﻿Results

### ﻿Molecular phylogenetic results

**Figure 1.**
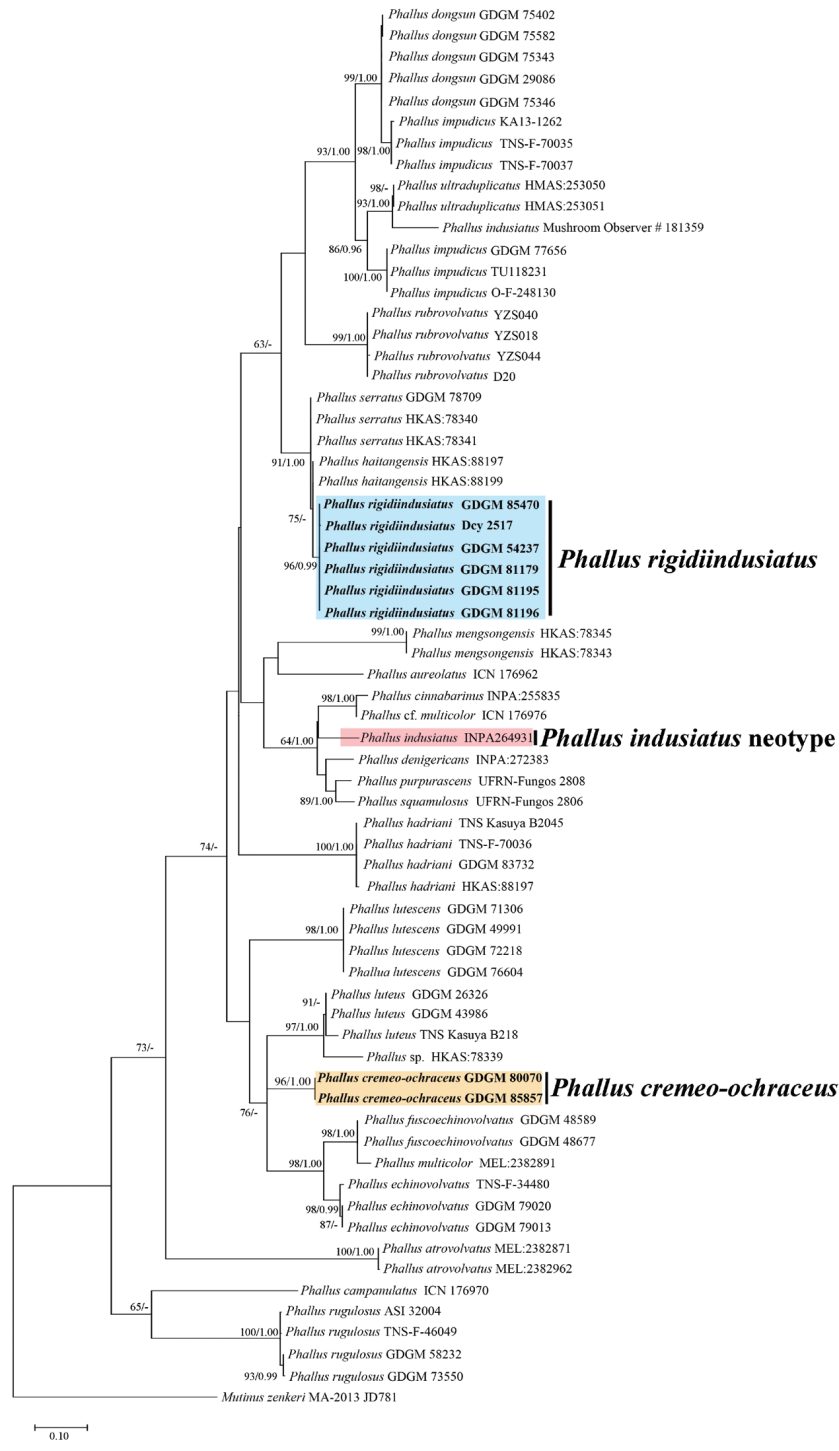
Phylogenetic overview of the genus *Phallus* inferred from ITS data using Maximum Likelihood (ML) and Bayesian Inference (BI). *Mutinuszenkeri* was selected as outgroup. Bootstrap values (≥50%) and Posterior probabilities (≥0.95) were presented around the branches.

**Figure 2.**
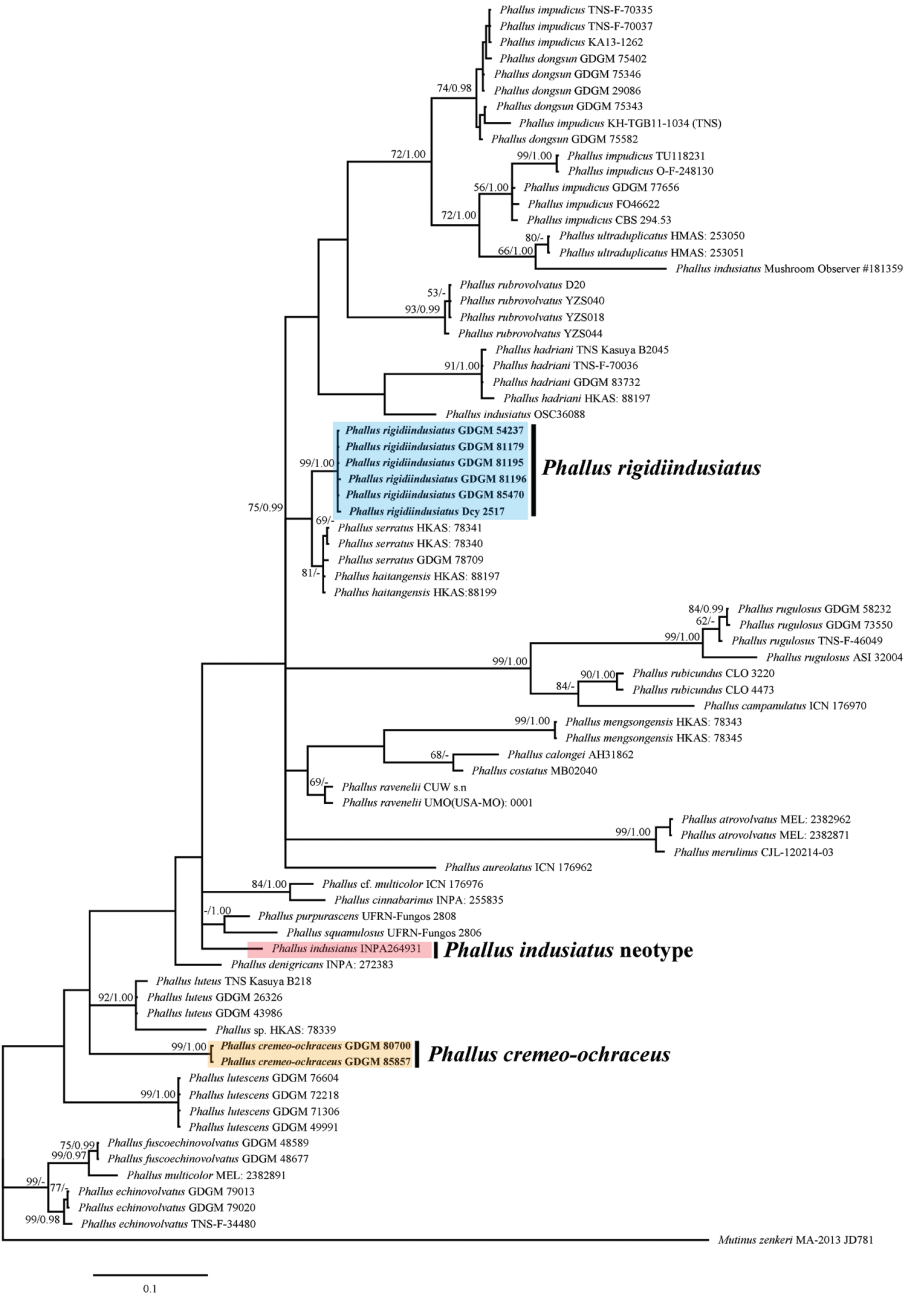
Phylogenetic overview of the genus *Phallus* inferred from concatenated data (ITS-LSU) using Maximum Likelihood (ML) and Bayesian Inference (BI). *Mutinuszenkeri* was selected as outgroup. Bootstrap values (≥50%) and Posterior probabilities (≥0.95) were presented around the branches.

In this study, sixteen sequences were newly generated from specimens of *Phallus* spp. and deposited in GenBank (Table 1), all of which were collected from China. In phylogenetic analyses, ITS dataset included 66 sequences from 27 taxa; ITS-LSU concatenated dataset included 77 assembled sequences consisting of 32 taxa; *Mutinuszenkeri* (Henn.) E. Fisch. (1900) was chosen as the outgroup (ITS: KC128650; LSU: KC128654) (Table 1). The ITS dataset contained 771 nucleotide sites (gaps included), and the concatenated dataset (ITS-LSU) contained 1687 nucleotide sites (gaps included) for each sample, of which 766 were ITS, 921 were LSU. In MrBays analyses, BI generations reached 3,458,000 for ITS dataset and 13,007,000 for ITS-LSU dataset as the value of stoprule became to 0.01, and the number of burn-in was 864.5 and 3251.75, respectively. Both ML and BI analyses had a very similar topological structure, but differed in minimum support values. Six collections (GDGM 54237, GDGM 81179, GDGM 81195, GDGM 81196, GDGM 85470 and Dcy 2517) are nested in a paraphyletic group containing *P.serratus* and *P.haitangensis* with strong supports (91%/1.00 BS/PP, Figure 1; 75%/0.99 BS/PP, Figure 2); while two other collections (GDGM 80700 and GDGM 85857), formed a monophyletic group containing *P.luteus*, *P.fuscoechinovolvatus*, *P.multicolor*, *P.echinovolvatus* with moderate supports in the ML analysis (76%/- BS/PP, Figure 1). However, in the ITS-LSU dataset analysis, both GDGM 80700 and GDGM 85857 separate from them and formed an independent clade with strong supports (99%/1.00 BS/PP, Figure 2).

**Table 1.** Sequences information of samples used for the ITS and ITS-LSU combined tree. Newly generated sequences were bold. The star “*” indicates the holotype or neotype specimens.

Name of the speices	Voucher/collection no.	Locality	LSU	ITS
*Phallusatrovolvatus*	MEL:2382871	Australia	KP012745	KP012745
*P.atrovolvatus*	MEL:2382962	Australia	KP012823	KP012823
*P.aureolatus*	ICN 176962*	Brazil	MF372127	MF372135
*P.calongei*	AH31862	Pakistan	FJ785522	–
*P.campanulatus*	ICN 176970	Brazil	MF372130	MF372138
*P.cinnabarinus*	INPA:255835	–	–	KJ764821
*P.costatus*	MB02040	–	DQ218513	–
***P.cremeo-ochraceus***	GDGM 80070*	**China**	**MZ890577**	**MZ890332**
***P.cremeo-ochraceus***	**GDGM 85857**	**China**	**MZ890578**	**MZ890333**
*P.denigricans*	INPA:272383*	Brazil	MG678455	MG678486
*P.dongsun*	GDGM 29086	China	MN264676	MN303794
*P.dongsun*	GDGM 75343	China	MN264678	MN303796
*P.dongsun*	GDGM 75346	China	MN264677	MN303795
*P.dongsun*	GDGM 75402*	China	MN264679	MN303797
*P.dongsun*	GDGM 75582	China	MN264680	MN303798
*P.echinovolvatus*	TNS-F-34480	Thailand	MF372129	MF372137
*P.echinovolvatus*	GDGM 79020	China	–	MN523216
*P.echinovolvatus*	GDGM 79013	China	MN611444	MN613536
*P.fuscoechinovolvatus*	GDGM 48589*	China	MF039585	MF039581
*P.fuscoechinovolvatus*	GDGM 48677	China	MF039586	MF039583
*P.hadriani*	OSC KH 11092003-1 Reference material	–	NG_060067	NR_119579
*P.hadriani*	TNS Kasuya B2045	Japan	KP222544	KP222542
*P.hadriani*	TNS-F-70036	Japan	KU516107	KU516100
*P.hadriani*	GDGM 83732	China	MW031865	MW031862
*P.haitangensis*	HKAS:88197*	China	–	NR_155668
*P.haitangensis*	HKAS:88199	China	–	KU705384
*P.impudicus*	CBS 294.53	U.K.	MH868748	–
*P.impudicus*	FO 46622	Germany	AY152404	–
*P.impudicus*	GDGM 77656	North Macedonia	MN264675	MN303793
*P.impudicus*	TU118231	Estonia	–	UDB015413
*P.impudicus*	O-F-248130	Norway	–	UDB038029
*P.impudicus*	KA13-1262	South Korea	–	KR673719
*P.impudicus*	TNS-F-70035	Japan	KU516106	KU516099
*P.impudicus*	TNS-F-70037	Japan	KU516108	KU516101
*P.impudicus*	KH-TGB11-1034 (TNS)	Japan	KF783249	–
*P.indusiatus*	Mushroom Observer # 181359	Mexico	–	MF428417
*P.indusiatus*	OSC36088	Japan	DQ218627	–
*P.indusiatus*	INPA264931*	Brazil	MG678463	MG678502
*P.lutescens*	GDGM 49991	China	MN131077	MN131081
*P.lutescens*	GDGM 71306	China	MN131074	MN131080
*P.lutescens*	GDGM 72218*	China	NG_073753	NR_171847
*P.lutescens*	GDGM 76604	China	MN131076	MN131078
*P.luteus*	TNS Kasuya B218	Japan	KP222545	KP222543
*P.luteus*	GDGM 26326	China	MT261793	MT261850
*P.luteus*	GDGM 43986	China	MT261794	MT261851
*P.mengsongensis*	HKAS:78345	China	–	KF052625
*P.mengsongensis*	HKAS:78343*	China	–	NR_158805
*P.merulinus*	CJL-120214-03	Guiana	KF783250	–
*P.multicolor*	MEL:2382891	Australia	KP012762	KP012762
P.cf.multicolor	ICN 176976	Guiana	MF372128	MF372136
*P.purpurascens*	UFRN-Fungos 2808*	Brazil	MG678456	MG678487
*P.ravenelii*	UMO(USA-MO):0001	USA	KP779906	–
*P.ravenelii*	CUW s.n	–	DQ218515	–
***P.rigidiindusiatus***	**GDGM 54237**	**China**	**MZ890579**	**MZ890334**
***P.rigidiindusiatus***	**GDGM 81179**	**China**	**MZ890580**	**MZ890335**
***P.rigidiindusiatus***	**GDGM 81195**	**China**	**MZ890581**	**MZ890336**
***P.rigidiindusiatus***	GDGM 81196*	**China**	**MZ890582**	**MZ890337**
***P.rigidiindusiatus***	**GDGM 85470**	**China**	**MZ890583**	**MZ890338**
***P.rigidiindusiatus***	**Dcy 2517**	**China**	**MZ890584**	**MZ890339**
*P.rubicundus*	CLO 3220	USA	MK652718	–
*P.rubicundus*	CLO 4473	USA	MK652720	–
*P.rubrovolvatus*	D20	China	–	MH381785
*P.rubrovolvatus*	YZS040	China	–	KF939503
*P.rubrovolvatus*	YZS018	China	–	KF939513
*P.rubrovolvatus*	YZS044	China	–	KF939515
*P.rugulosus*	TNS-F-46049	China, Taiwan	MF372134	MF372142
*P.rugulosus*	ASI 32004	-	-	AF324169
*P.rugulosus*	GDGM 58232	China	MT261858	MT361864
*P.rugulosus*	GDGM 73550	China	MT261859	MT361865
*P.serratus*	HKAS:78341	China	–	KF052623
*P.serratus*	HKAS:78340*	China	–	KF052622
*P.serratus*	GDGM 78709	China	MZ508445	MZ508443
*P.squamulosus*	UFRN-Fungos 2806*	Brazil	–	MG678497
*P.ultraduplicatus*	HMAS:253050*	China	KJ591586	KJ591584
*P.ultraduplicatus*	HMAS:253051	China	KJ591587	KJ591585
*P.* sp.	HKAS:78339	China	–	KF052621
*Mutinuszenkeri*	MA-2013 JD781	São Tomé and Principe (Africa)	KC128654	KC128650

## ﻿Taxonomy

### ﻿ Phallus cremeo-ochraceus

Taxon classificationFungiPhallalesPhallaceae

 T. Li, T.H. Li & W.Q. Deng sp. nov.

7487B0A1-61F7-5609-93A5-59A261F5EE62

MycoBank No: 840963

Figures 3 , 5a–c

#### Diagnosis.

Similar to *Phallusindusiatus* with an indusium almost touching ground, but mainly characterized by the cream to ochraceous receptacle, white to very slightly pinkish indusium and pseudostipe, white to pinkish volva, and basidiospores up to 4.0 × 1.7 µm.

**Figure 3.**
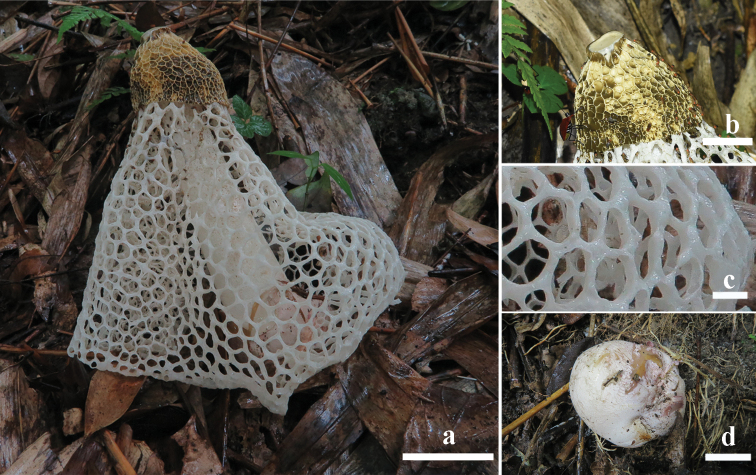
Basidiomata of *Phalluscremeo-ochraceus***a-c**GDGM 80700 **d**GDGM 85857. Scale bars: 5 cm (**a**), 2 cm (**b, d**), 1 cm (**c**).

***Holotype***. China. Guizhou Province, Libo County, Xiaoqikong Scenic Area (25°15'12"N, 107°44'16"E, alt. 428 m), Zhang Ming, 2 July 2020 (GDGM 80700).

Immature basidioma globose to subglobose, 55 × 50 mm, white to pinkish (9A2), purplish pink (14A4) when injured, smooth to very slightly rimose-areolate, attached to substrate by pinkish white to pinkish (9A2) rhizomorphs. Exoperidium membranous; endoperidium gelatinous, hyaline. Expanded basidioma up to 240 mm high when fresh. Receptacle 42–50 mm high, 50–60 mm broad, campanulate, cream to ochraceous (4A3-5), reticulated with irregularly ridges up to 4.0 mm deep, covered with gleba; apex truncate, with a pale yellow (4A2), prominent disc up to 15 mm in diam. Gleba olive brown (4E4-6, 4F5-8), mucilaginous. Pseudostipe subcylindrical, constricted at apex, enlarged downwards, 200–220 mm high when mature, 22–27/32–38/40–45 mm broad (apex/middle/base), white (9A1) to slightly pinkish white (9A2), spongiform, hollow; pseudostipe wall 6–9 mm thick, usually consisting of small irregular chambers up to 3 mm. Volva obovate, 47–52 mm high, 40–45 mm broad, smooth, pinkish (9A2). Indusium well-developed, almost touching ground, white to very slightly pinkish, 190–210 mm in length, attached to the apex of pseudostipe, with polygonal to irregular meshes; meshes 7–20 mm wide, 2–4 mm thick. Rhizomorphs simple, yellowish white (4A2) to pinkish (9A2), 1–2 mm thick, about 20 mm long. Odour foetid (mainly from gleba). Taste mild.

Basidiospores (3.2–)3.5–3.8(–4.0) × 1.2–1.5(–1.7) μm, Q= (2.0–)2.3–2.7(–3.0), Q_m_= 2.5 ± 0.5, cylindrical to long ellipsoid, hyaline and light olivaceous in H_2_O and 5% KOH solution, inamyloid, thin-walled, smooth under light microscope. Hyphae of receptacle, pseudostipe and indusium hyaline or slightly yellowish, thin-walled, pseudoparenchymatic, consisting of globose to subglobose or irregularly globose cells up to 30 μm in diam. Hyphae of volva tubular and branched, 4–8 μm in diam., thin-walled, smooth, septate, with clamp-connections. Hyphae of rhizomorphs filamentous, up to 8.0 μm in diam., thin-walled, smooth, septate, rarely branched.

#### Habitat and distribution.

Solitary or scattered on soil with decaying litter under bamboo groves. So far known only from southwestern China (Guizhou). Season: July.

#### Etymology.

With reference to the cream to ochraceous color of receptacle.

#### Additional specimens examined.

China. Guizhou Province, Libo county, Xiaoqikong Scenic Area (25°15'46"N, 107°41'4"E, alt. 480 m), Zhang Ming, 2 July 2020, (GDGM 85857).

### ﻿ Phallus rigidiindusiatus

Taxon classificationFungiPhallalesPhallaceae

T. Li, T.H. Li & W.Q. Deng sp. nov.

9D120549-24DC-56C1-B213-A78C55784046

MycoBank No: 840965

Figures 4 , 5d–f

#### Diagnosis.

Characterized by a well-developed indusium with thick meshes, morphologically similar to *Phallusserratus*, but different in its rigid, round or irregular meshes of indusium without serrated margin, and in smaller basidiospores.

**Figure 4.**
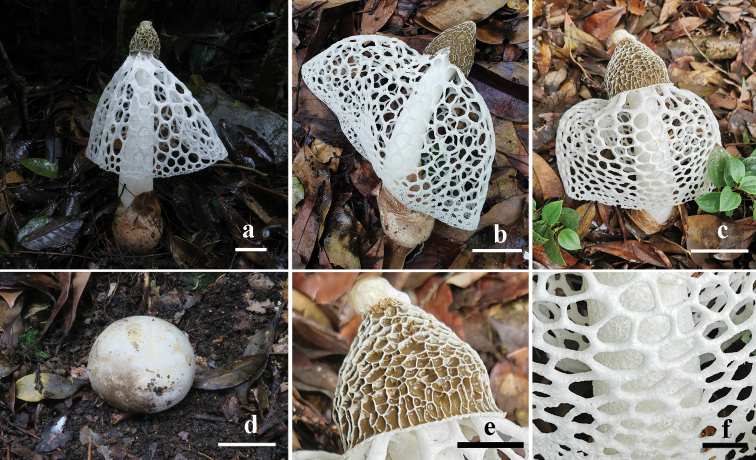
Basidiomata of *Phallusrigidiindusiatus*. **a**GDGM 54237 **b**GDGM 85470 **c, e, f**GDGM 81196 **d** 81195. Scale bars: 5 cm (**a-c**), 3 cm (**d**), 2 cm (**e**), 1 cm (**f**).

**Figure 5.**
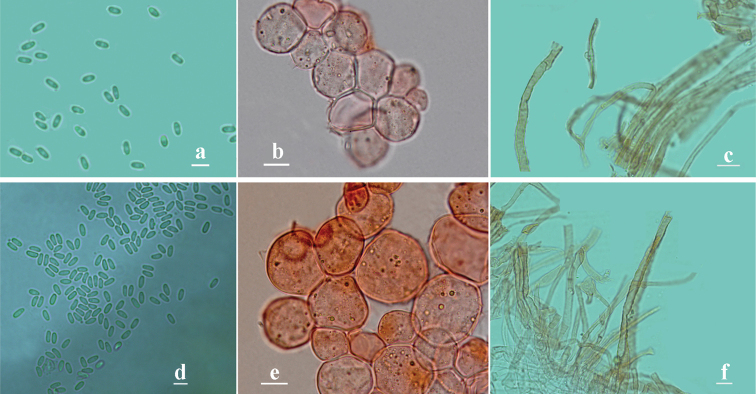
Characteristics of *Phalluscremeo-ochraceus***a-c** and *Phallusrigidiindusiatus***d-e** under the light microscope. **a, d** basidiospores **b, e** pseudoparenchymatous hyphae from pseudostipe **c, f** hyphae from volva. Scale bars 5 µm (**a–f**).

***Holotype.*** China. Guangdong Province, Jiangmen City, Yunkaishan National Nature Reserve. (22°17'57"N, 111°12'37"E, alt. 1350 m), Song Bin and Wen Huashu,10 June 2020 (GDGM 81196).

Immature basidioma globose to subglobose, 55–65 × 50–57 mm, white (1A1), slightly yellowish white (4A2) to orange white (7A2) or pinkish white (10A2), partially darker to grayish brown (7D3), smooth, attached to substrate by grayish violet (17D5-7) rhizomorphs. Exoperidium membranous; endoperidium gelatinous, hyaline. Expanded basidioma big-sized, 220–240 mm high when fresh. Receptacle 40–50 mm high, 50–60 mm broad, campanulate to subconical, white (1A1) to yellowish white (3A2), reticulated with irregularly ridges up to 4.5 mm deep, covered with gleba; apex truncate, perforated, or with a white spongy expansion up to 8 mm high, 10 mm in diam. Gleba yellowish brown to linoleum brown (5E5-7), mucilaginous. Pseudostipe subcylindrical, constricted at apex, enlarged toward base, white (1A1), spongiform, hollow, 170–190 mm high, 15–20/28–35/35–40 mm broad (apex/middle/base); pseudostipe wall 5–9 mm thick, usually consisting of small irregular chambers in 1–3 mm width. Volva obovate, 55–65 mm high, 50–60 mm broad, smooth, brownish orange (7C6) to light brown (7D8). Indusium well-developed, expanded to 3/4–5/6 portion of pseudostipe, white, up to 170 mm in length, attached to apex of pseudostipe, with rigid polygonal to irregular meshes becoming gradually smaller from top to bottom, margin entire; meshes usually not serrated at margin, 5–20 mm wide, up to 7 mm thick. Rhizomorphs simple, grayish orange (6C5) to brown (7E4), up to 3 mm thick, 4 cm long. Odour foetid (mainly from gleba). Taste mild.

Basidiospores (3.5–)3.7–4.2(–4.5) × 1.6–2.0(–2.3) μm, Q= (1.7–)2.1–2.4 (–2.6), Q_m_= 2.3 ± 0.2, cylindrical to long ellipsoid, hyaline and light olivaceous in H_2_O and 5% KOH solution, inamyloid, thin-walled, smooth, truncate at one end under light microscope. Hyphae of receptacle, pseudostipe and indusium hyaline, thin-walled, pseudoparenchymatic, consisting of globose to subglobose or irregularly globose structures, up to 25 μm in diam. Hyphae of volva tubular and branched, 3–5 μm in diam., thin-walled, smooth, septate, with clamp-connections. Hyphae of rhizomorphs filamentous, up to 6.0 μm in diam., thin-walled, smooth, septate, rarely branched.

#### Habitat and distribution.

Solitary or scattered on soil with decaying litter in forests dominated by broad-leaved trees and bamboo groves. So far known only from southern China and southwestern China (Guizhou). Season: May to June.

#### Etymology.

With reference to the rigid indusium.

#### Additional specimens examined.

China. Hunan Province, Rucheng County, Jiulongjiang National Forest Park (25°26'49"N, 113°48'10"E, alt. 555 m), Huang Hao, 7 May 2015 (GDGM 54237); Guizhou Province, Duyun County, Doupengshan scenic place (26°21'17"N, 107°22'49"E, alt. 1300 m), Deng Chunying, 16 May 2020 (Dcy2517); Guangdong Province, Shaoguan City, Nanling National Nature Reserve (24°49'54"N, 113°7'22"E, alt. 994 m), Song Bin and Xie Dechun, 27 May 2021 (GDGM 85470); Guangdong Province, Jiangmen City, Yunkaishan National Nature Reserve. (22°15'22"N, 111°9'23"E, alt. 1480 m), Song Bin and Wen Huashu, 10 June 2020 (GDGM 81179); Guangdong Province, Jiangmen City, Yunkaishan National Nature Reserve. (22°17'58"N, 111°12'36"E, alt. 1420 m), Song Bin and Wen Huashu, 10 June 2020 (GDGM 81195).

## ﻿Discussion

Based on the ITS dataset *P.cremeo-ochraceus* nested in a group containing *P.luteus*, *P.echinovolvatus*, *P.fuscoechinovolvatus* and *P.multicolor* (Figure 1). However, in the ITS-LSU dataset *P.cremeo-ochraceus* separates from them and formed an independent clade (Figure 2). Therefore, the sister relationships of *P.cremeo-ochraceus* remain unclear. Morphologically, all of them have similar color in receptacle except *P.multicolor* and *P.luteus* which have a bright yellow to orange indusium (Berkeley and Broome 1883; Kasuya 2008).

Phylogenetically, *P.rigidiindusiatus* is closely related to *P.serratus* and *P.haitangensis* with strong support (Figures 1, 2). Morphologically, *P.serratus* resembles *P.rigidiindusiatus* in having a white and strongly reticulate receptacle, a white and well-developed indusium and a brownish-gray volva. However, *P.serratus* can be easily distinguished from the new species in having the serrated meshes of indusium and larger basidiospores (4–5 × 2–3 µm) (Li et al. 2014); *Phallushaitangensis* is another closely related taxon, which is different in its golden orange receptacle and a well-developed, light orange indusium (Li et al. 2016). Interestingly, *P.haitangensis* and *P.serratus* have distinct morphological characteristics but shared with a 98.4% similarity of ITS sequence (Li et al. 2014, 2016). Both two new species were separated from *P.indusiatus* in phylogenetical analyses.

Other *Phallus* species with a white indusium are relatively easier to be distinguished from the new species *P.cremeo-ochraceus* and *P.rigidiindusiatus* (Table 2). For example, the Chinese species *P.echinovolvatus* and *P.fuscoechinovolvatus* are distinguished by having an obviously echinate volva (Zang et al. 1988; Song et al. 2018); and *P.atrovolvatus* Kreisel & Calonge, described from the Central America, can be easily distinguished by having a rugulose to merulioid receptacle, a black volva, and an indusium expanded to midway from the receptacle and volva (Calonge 2005). Although the Brazilian species *P.aureolatus* L. Trierveiler-Pereira & A.A.R. de Meijer has a rigid, white and almost touching ground indusium which is similar to that of *P.rigidiindusiatus*, it differs in having a rugulose to merulioid receptacle, a shorter pseudostipe (up to 10 cm high) and a shorter basidiospores (3.0–4.1 × 1.5–2.0 μm) (Trierveiler-Pereira et al. 2017).

**Table 2.** Type location, receptacle, volva, indusium, and basidiospores of the *Phallusindusiatus*-like species.

Species name	Type location	Receptacle	Volva	Indusium	Basidiospores
***Phalluscremeo-ochraceus***	China, Guizhou	Pale yellow to light yellow, reticulated	Pinkish, smooth surface	Almost touching the ground	3.2–4.0×1.2–1.7 μm
***P.echinovolvatus***	China, Hunan	White to yellow, reticulated	Whitish or pale brown, with echinulate projections	Almost touching the ground	3.0–4.0×1.3–2.0 μm
***P.fuscoechinovolvatus***	China, Guangdong	Yellowish, reticulated	Dark brown or blackish, with many white to pale yellow echinules	Almost touching the ground	2.5–4.0×1.0–2.0 μm
***P.indusiatus***	Brazil, Pará	White, reticulated	White, with pinkish pigments	Extending to the ground	3.6–4.1×1.5–2.2 µm
***P.merulinus***	Indonesia, Java	White, minutely convoluted folds	Dull white	Expanded to 1/2 portion of pseudostipe	3.3–4.0×1.4–1.8 μm
***P.rigidiindusiatus***	Southern and Southwestern of China	White to yellowish, reticulated	Brownish orange to light brown, smooth surface	Expanded to 3/4–5/6 portion of pseudostipe, with rigid polygonal to irregular meshes, without serrated margin.	3.5–4.5×1.6–2.3 μm
***P.rubrovolvatus***	China, Yunnan	Yellowish, reticulated	Dark purple, smooth surface	Expanded to 1/2 portion of pseudostipe	3.7–4.0×1.5–2.5 μm
***P.serratus***	China, Yunnan	White, reticulated	Brownish-gray, without scales	Almost touching the ground, with the serrated margin in hole of indusium.	4.0–5.0×2.0–3.0 µm
***P.ultraduplicatus***	China, Liaoning	White, reticulated	Flesh-ocher	Short, 20–40 mm long,	4.0–5.0×1.5–2.0 µm

Among the complex members of *P.indusiatus* s.l. published by Cabral et al. (2019), *P.denigricans* T.S. Cabral, B.D.B. Silva & Baseia has a volva varying from white to dark brown and basidiospores up to 4.6 × 2.5 µm; *Phalluspurpurascens* T.S. Cabral, B.D.B. Silva & Baseia has a white receptacle, a purplish volva and larger basidiospores (4.4–5 × 2.5–3.4 µm); and *P.squamulosus* T.S. Cabral, B.D.B. Silva & Baseia is characterized by its squamous surfaces of immature basidioma and volva. Besides, *P.maderensis* Calonge, described from the Atlantic Island of Africa, has an interesting indusium attaching to the base of pseudostipe and is not hanging from the receptacle (Calonge et al. 2008); and *P.merulinus* (Berk.) Cooke from Indonesia differs in a rugose receptacle with minutely convoluted folds (Lloyd 1909). The Chinese species *P.rubrovolvatus* is distinguished by the red purple volva, although it also has a rigid indusium reaching on the midway or 3/4 portion of the pseudostipe (Liu et al. 2005); and *P.ultraduplicatus* X.D. Yu, W. Lv, S.X. Lv, Xu H. Chen & Qin Wang from northeastern China has a shorter indusium hanging down less than 1/2 portion of the pseudostipe and longer and narrower basidiospores than those of *P.rigidiindusiatus* (Adamčík et al. 2015).

According to the original description, *Phallusindusiatus*, a South American species, is characterized by the campanulate and reticulated receptacle and the white indusium touching the ground (Ventenat 1798). However, it was not possible to find the original material in herbarium for comparison due to the unspecific information (Ventenat 1798). Recently, based on same characteristics as the original description, close geographical location with the same forest domain, and submitted the available molecular sequences to GenBank, a neotype of *P.indusiatus* was designated, which has a campanulate and reticulated receptacle, a white and fully developed indusium, a white volva and elongated and smooth basidiospores (3.6–4.1 × 1.5–2.2 µm); according to all known data about the *Phallus* taxa, its distribution is presumed to be restricted to South America (Cabral et al. 2019).

In phalloid fungi, macro-characters, such as the shape, the surface characters and color of the main structures (receptacle, pseudostipe, indusium, volva and rhizomorphs), are generally more important than micro-characters for infrageneric classification (Kreisel 1996). Therefore, if without any molecular phylogenetic analyses, two or more species shared similar macro-characters, then these could easily be confused for the same species. However, when geographical distribution has been taken into account as the taxonomic evidence, they tend to become easily distinguishable, because phalloid fungi have a passive basidiospore dispersal mechanism that depends mainly on insects as transporters, and this factor together with environmental conditions (such as temperature, humidity, illumination, soil nutrition and dominated plants) arguably limit their geographical distributions (Wilson et al. 2011). According to our previous studies, for example, quite a lot of Asian specimens labeled as “*P.impudicus*” were actually identical to *P.dongsun* from China, and *Phallusrubicundus* (Bosc) Fr. originally described from America was probably not naturally distributed in China, even in Asia (Li et al. 2020a, b). Therefore, morphological analyses and geographical distributions, as well as molecular phylogeny are the most useful evidences to identify the phalloid fungi. The two *Phallusindusiatus*-like species from China were proven as new to science with strong supports of those evidences in this study while the natural distribution of *P.indusiatus* in China becomes more suspicious.

### ﻿Key to *Phallus* species with a white or nearly white indusium

**Table:** 

1	Volva squamulose or echinulate	**2**
–	Volva smooth or nearly so, not squamulose or echinulate	**4**
2	Volva surface squamulose, white	***P.squamulosus***
–	Volva surface obviously echinulate	**3**
3	Volva dark brown or blackish	***P.fuscoechinovolvatus***
–	Volva generally white	***P.echinovolvatus***
4	Volva discoloring from white to dark brown	***P.denigricans***
–	Volva unchanging in color or only slightly discoloring, not discoloring to dark brown	**5**
5	Receptacle rugulose to merulioid	**6**
–	Receptacle reticulate	**8**
6	Volva black	***P.atrovolvatus***
–	Volva pinkish or white	**7**
7	Vovla pinkish; indusium almost touching ground	***P.aureolatus***
–	Volva white, with minutely convoluted folds; indusium not touching ground	***P.merulinus***
8	Indusium attached to the base of the pseudostipe and free from receptacle	***P.maderensis***
–	Indusium attached to the apex of the pseudostipe	**9**
9	Volva white	***P.indusiatus***
–	Volva colored	**10**
10	Indusium shorter than 40 mm when mature	***P.ultraduplicatus***
–	Indusium longer than 40 mm when mature	**11**
11	Receptacle cream to ochreous	***P.cremeo-ochraceus***
–	Receptacle white	**12**
12	Indusium with obviously serrated meshes	***P.serratus***
–	Indusium with round or irregular meshes, but without obviously serrated meshes	**13**
13	Volva brownish orange to light brown, not red to purple obviously; indusium strongly rigid; basidiospores narrower, (3.5–)3.7–4.2(–4.5) × 1.6–2.0(–2.3) μm	***P.rigidiindusiatus***
–	Volva obviously red to purple; basidiospores broader	**14**
14	Volva deep red; basidiospores smaller, 3.7–4 × 2–2.5 µm	***P.rubrovolvatus***
–	Volva purplish or becoming purple; basidiospores larger, 4.4–5 × 2.5–3.4 µm	***P.purpurascens***

## Supplementary Material

XML Treatment for Phallus cremeo-ochraceus

XML Treatment for Phallus rigidiindusiatus
